# Personality Disorders in Criminal Offenders - A Systematic Literature Review

**DOI:** 10.1007/s11920-024-01541-0

**Published:** 2024-10-16

**Authors:** Aleya Flechsenhar, Sarah Back, Max Knabe, Katja Bertsch

**Affiliations:** 1 Department of Psychology, Ludwig-Maximilians-Universität München, München, Germany; 2 Department of Psychology, Julius-Maximilians-Universität Würzburg, Wuerzburg, Germany; 3 German Center for Mental Health (DZPG), partner site München/Augsburg, München, Germany

**Keywords:** Personality disorders, Alternative model, DSM-5 AMPD, ICD-11, Offenders in forensic-psychiatric treatment or prison

## Abstract

**Purpose of Review:**

We summarized studies investigating measures related to the Alternative Model for Personality Disorders (AMPD) of the DSM-5 and the personality model in ICD-11 in offenders in forensic-psychiatric treatment or prison to evaluate its forensic utility.

**Recent Findings:**

The reformation of the DSM and ICD with regards to the introduction of dimensional assessments of personality disorders holds many advantages over categorical models concerning clinical utility.

**Summary:**

With regards to DSM-5 AMPD *Criterion A*, a limited number of studies (*k* = 4) report impairments in interpersonal functioning in offenders. Studies assessing *Criterion B* (*k* = 13) predominantly report higher personality impairment measures for offenders, especially for antagonism and disinhibition. Due to the heterogeneity of the selected studies, this review cannot draw conclusions with regard to the predictive value of dimensional models for offenders in forensic-psychiatric treatment or prison, but provides initial evidence for the validity and utility of DSM-5 AMPD and ICD-11 in these settings.

## Introduction

The association between personality functioning and offending has been previously studied, in particular with regard to personality disorders (see reviews of [1, 2]). Personality functioning (i.e., personality psychopathology, such as *identity*, *self-direction*, *empathy*, and *intimacy* [3]) interacts with other risk factors (e.g., comorbid mood disorders, substance abuse, aggression, maladaptive cognitions and beliefs) to contribute to offending behavior [4] and indicates treatment completion for personality-disordered offenders [5]. Whereas classification systems of previous versions of the ICD (International Statistical Classification of Diseases and Related Health Problems) or DSM (Diagnostic Statistical Manual) follow categorical approaches for the diagnosis of mental disorders, the ICD-11 and DSM-5 have recently added a dimensional approach, which account for the notion that pathology exists on a spectrum rather than a dichotomy. The introduction of this new approach has, however, led to controversial opinions across different fields of psychological research and practice. Especially with regard to the diagnosis of personality disorders, the categorical models show several caveats. The ICD-11 and the Alternative Model of Personality Disorders (AMPD) of the DSM-5 provide a scientifically derived approach to diagnosing personality disorders to address the caveats of the established personality disorder categories in DSM-5 and ICD-10 [6]. These novel approaches offer markers of severity for each facet or impairment (mild, moderate, severe) with optional characterization of prominent maladaptive traits or trait qualifiers, and further allow for assigning categories (DSM-5 AMPD) or patterns (ICD-11) of personality. This is in contrast to conventional categorical models, whose insufficient reliability, prognostic validity, and handling of comorbidities, has been questioned. Even though doubts remain with regard to the utility of these approaches, its clinical value has been highlighted in several studies (e.g [6, 7]), however, there is no overview of its utility in forensic settings. This is of interest since aggression, that is, the extent to which a person can modulate the experiences and expression of aggression, or is dominated by aggression, has already been regarded as an important additional dimension of personality pathology, particularly in those individuals with high levels of impairments in Kernberg’s model of personality organization (1984) [8]. The link between (impaired) personality organization and aggression has been confirmed in several empirical studies (e.g [9]).

Previous research using the traditional categorical assessment approach to personality disorder has established that a large proportion of male offenders convicted of violent offending, or who have a history of aggression, meet criteria for a minimum of one personality disorder, with rates as high as 62–84% [10, 11]. Recent evidence using dimensional models also indicates associations between personality disorders and aggression and violent behavior [9, 12]. This research also considers other new models, such as the concepts of *personality structure* and *personality organization* (for review see [3]), which are considered pivotal concepts that substantially influenced the development of personality functioning [13]. High emotional reactivity or the lack of empathy in the forensic context are prevalent and highlight the importance of their consideration in offenders [14, 15]. According to the ICD-10, personality disorders manifest in childhood and early adolescence and remain relatively stable of the course of life. Empirical research, however, suggests that rates of remission differ greatly as a function of personality disorder [16–18]. The ICD-11 now refrains from specifying a minimum age limit for the diagnosis of personality disorders and instead presupposes a duration of symptoms for at least two years as criterion of stability. This lack of temporal restriction is one of the reasons why forensic-psychiatric assessments show restraint in integrating dimensional approaches in forensic settings. From a clinical perspective, individuals are able to receive treatment earlier, possibly before manifestation of the disorder. From a forensic perspective, young individuals may receive unlimited forensic placement, which in turn may cause more restrictive diagnosing to prevent such sentences. Forensic psychiatrists also fear that this criterion may lead to a higher number of personality disorder diagnoses, also in juvenile cases, or in cases of symptom remission after hospitalization (as opposed to forensic commitment, or imprisonment). This risk underscores the need for special awareness of the problem in the forensic context regarding the determination of severity in the context of culpability. This problem is further emphasized by the fact that legal questions for the assessment of culpability often need to be answered dichotomously (i.e., does the diagnosis of the personality disorder correspond to the characteristic of *severe other mental abnormality* [19]). Nevertheless, a lack of clear delineation of psychopathologies makes relevant assessments difficult and diagnoses whose reliability over time are questionable hardly meet the strict requirements for the special accommodation of mentally disordered offenders.

Therefore, it is important to investigate and understand dimensional models of the DSM-5 AMPD and the ICD-11 in forensic research and practice. This systematic review aims to summarize empirical studies investigating forensic samples (i.e., currently incarcerated prisoners or forensic-psychiatric patients). While there have been reviews on the utility of the AMPD within a clinical population (e.g [20]), similar assessments with forensic populations are lacking.

## Methods

The *Pubmed*, *Web of Science* and *APA PsychNET* databases were utilized to receive empirical, peer-reviewed, English language studies investigating dimensional personality pathology assessments in criminal offenders compared to control samples. We included all studies published since 2013 that measured and used official instruments for this purpose of personality pathology according to the conceptual models of DSM-5 AMPD or ICD-11.

The reporting of this systematic review was guided by the standards of the Preferred Reporting Items for Systematic Review and Meta-Analysis (PRISMA) Statement [21].

### Search Strategy

Three different search engines (PubMed, *n* = 227; Web of Science, *n* = 294; APA PsycNet, *n* = 87) were used for the search with the following Boolean string:

(antagonism [Title/Abstract] OR disinhibition [Title/Abstract] OR “negative affectivity” [Title/Abstract] OR psychoticism [Title/Abstract] OR Detachment [Title/Abstract] OR anancas* OR AMPD[Title/Abstract] OR ICD-11[Title/Abstract] OR “maladaptive traits” [Title/Abstract] OR “personality functioning” [Title/Abstract]) AND (crim* [Title/Abstract] OR incarcerated [Title/Abstract] OR “criminal record” [Title/Abstract] OR misconduct [Title/Abstract] OR crime [Title/Abstract] OR convict* [Title/Abstract] OR offense [Title/Abstract])

## Data Extraction

The survey of literature according to these search terms the respective databases took place over ten months from January 2023 to October 2023. Subsequently, all the databases mentioned were systematically searched according to the scheme outlined above, all search hits were listed in a table. After the survey was completed, all duplicate search results were removed. A predefined scheme of inclusion and exclusion criteria was applied to the remaining studies. Data were extracted into a Microsoft Access database and checked by two authors separately. Conflicts were resolved by discussion and referred to a third reviewer if necessary. The information relevant to the research question of this thesis was extracted from the included studies and summarized in Table 1.

## Inclusion Criteria

Studies had to be peer-reviewed journals published in English and include a validated psychometric measure of personality functioning (or personality severity) and/or maladaptive traits (or trait domains) operationalized according to and on the basis of the DSM-5 AMPD in Section III and/or ICD-11 model. Further, the included sample is required to have a substantiated criminal record (e.g., current or previous incarceration either in prison, or in a forensic-psychiatric hospital). Study methodology needed to include a quantitative analysis of longitudinal, experimental, or cross-sectional data. Lastly, all studies needed ethical approval and ascertainment of written informed consent. Publication dates were restricted to 2013 and after in line with the issue of DSM-5 AMPD.

**Fig. 1:**
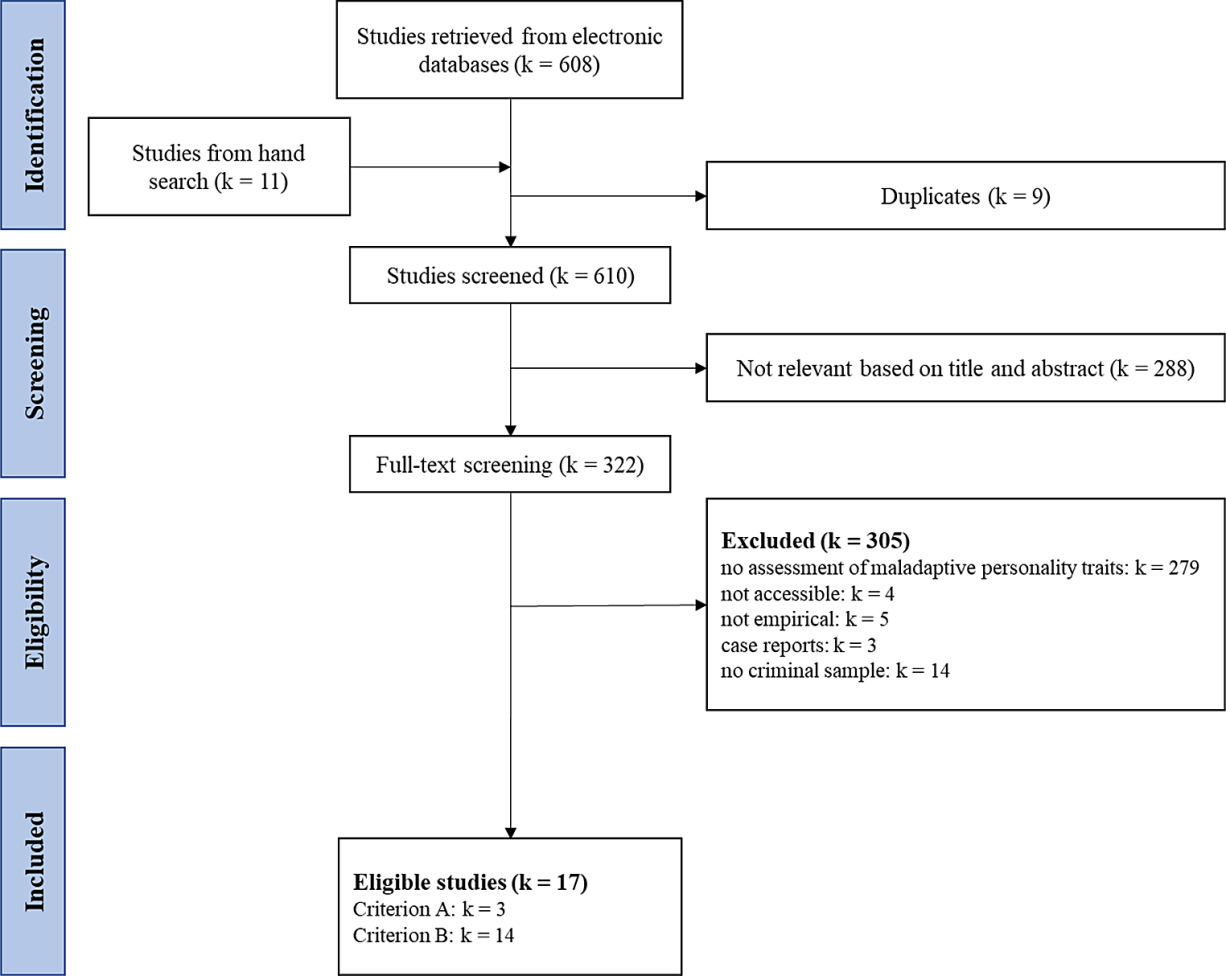
Flow chart to represent the selection process of this review based on the PRISMA 2020 criteria [22]

## Results

**Table 1:** Search results of eligible studies included (*n* = 17)

Authors	Measures of personality functioning	Criminal record	*n* (total)	Main results
Adhiatma & Halim (2016) [23]	Personality Inventory for DSM-5 (PID-5)	The participants in this study were categorized into incarcerated and non-incarcerated groups. Within the incarcerated group, there were subgroups of violent offenders (*n* = 96; males = 96, females = 0), non-violent offenders (*n* = 79; males = 77, females = 2) and drug offenders (*n* = 180; males = 159, females = 21).	96 violent offenders 79 non-violent offenders 180 drug offenders 245 non-prisoners	There were significant differences between groups for negative affectivity (*F*(3,596) = 9.57, *p* < .05), Antagonism (*F*(3,596) = 10.01, *p* < .05), detachment (*F*(3,596) = 32.58, *p* < .05), disinhibition (*F*(3,596) = 13.52, *p* < .05), psychoticism (*F*(3,596) = 10, *p* < .05). Post hoc analyses revealed that prisoners had higher scores respectively than non-prisoners.
Bach & Anderson (2020) [24]	Level of Personality Functioning Scale-Brief Form (LPFS-BF) Personality Inventory for DSM-5 Short Form (PID-5 SF) Young Schema Questionnaire Short Form 3 (YSQ-S3)	Psychiatric outpatients (males = 15, females = 50). Prisoners were recruited from a prison unit specialized in the treatment of personality disorder and substance/alcohol abuse (all males). This sample showed high scores on externalizing and antisocial features in comparison to the psychiatric outpatients.	65 psychiatric outpatients 85 offenders in prison (all males)	Mean total scores of the LPFS differed between outpatients and prisoners (Cohen’s *d* = 0.79, *p* < .05), but the PID-5 (total severity) did not. The PID-5 correlated with levels of personality functioning (*r* = .83, *p* < .001). Maladaptive schemas correlated with levels of personality functioning on all scores (disconnection and rejection, *r* = .79; impaired autonomy and performance, *r* = .75; excessive responsibility and standards, *r* = .60; and impaired limits, *r* = .63, all *p*’s < 0.001).
Bach & Hutsebaut (2018) [25]	Level of Personality Functioning Scale-Brief Form 2.0 (LPFS-BF 2.0) Personality Inventory Short Form (PID-5 SF) Schema Mode Inventory (SMI)	The incarcerated individuals were recruited from a prison unit specialized in treatment of cooccurring personality pathology and substance abuse (psychiatric outpatients males = 26; incarcerated patient males = 107).	122 psychiatric outpatients 107 incarcerated addict treatment patients	Outpatients showed a higher mean score on LPFS self-functioning relative to interpersonal functioning and showed substantially higher PID–5 scores on Negative affectivity in terms of submissiveness, anxiousness, emotional lability, and depressivity relative to the prisoners. On the other hand, the prisoners showed significantly higher PID-5 scores on antagonism (i.e., deceitfulness, manipulativeness, callousness, and grandiosity) along with facets of disinhibition (risk taking) and detachment (restricted affectivity) in comparison to the outpatients. There were no correlations between PID-5 total mean score and LPFS total score (*r* = .79), LPFS self-functioning (*r* = .69), and LPFS interpersonal functioning (*r* = .74; all *p*’s > 0.05).
Billen et al. (2023) [26]	Personality Inventory for DSM-5 (PID-5)	The study included forensic-psychiatric male patients from three high-security forensic-psychiatric centers in Belgium and the Netherlands.	94 forensic-psychiatric patients	Significant negative residual correlations were found between identity dysfunction and psychoticism (*r* = − .40; *r* = − .42) and a negative association between childhood trauma and antagonism (*r* = –.34), suggesting that more traumatic experiences were associated with less antagonistic behavior. For detachment, emotion regulation was the only significant predictor, and for all outcomes except antagonism, it was the strongest predictor, emphasizing the importance of the emotional aspect of self-regulation when it comes to clinical outcomes relevant in the forensic setting.
Boland et al. (2021) [27]	Personality Inventory for DSM-5 Brief Form (PID-5-BF)	Data was collected from 180 inmate participants (males = 114, females = 66) at a county jail in the southeastern United States through the distribution of recruitment flyers and sign-up sheets by jail staff across the entire correctional facility.	180 offenders in prison	All personality psychopathology variables were significantly associated with number of adult arrests (r’s = 0.20–0.25, p’s < 0.01), with the exception of disinhibition (*r* = .08, *p* = .26).
Dunne et al. (2020) [28]	Personality Inventory for DSM-5 (PID-5)	Participants were 208 males incarcerated in a remand prison setting in Australia.	208 offenders in prison	Partial correlations were found between life history of aggression and callousness (*r* = .32, *p* < .001), hostility (*r* = 48, *p* < .001), impulsivity (*r* = 30, *p* < .001), irresponsibility (*r* = .20, *p* < .01), Risk Taking (*r* = .32, *p* < .001), Withdrawal (*r* = .19, *p* < .01), and perseveration (*r* = .21, *p* < .01).
Dunne et al. (2021) [29]	Personality Inventory for DSM-5 Brief Form (PID-5-BF)	The intention was to recruit as many of the total inmate population as possible (all males). Data were collected using a three-stage data collection process. The first stage involved recruiting prisoners during educational and employment program time. The second recruitment stage involved researchers moving from unit to unit around the prison. The third stage involved collecting data during lock down.	438 offenders in prison	The PID-5-BF total score was found to be positively correlated with self-reported aggression (*r* = .42, *p* < .001), as were each of the PID-5-BF domain scores (r-values ranging from *r* = .19, *p* < .001 to *r* = .47, *p* < .001). Multiple regression analyses showed that the PID-5-BF total score was a significant predictor, explaining 17.5% (adjusted *R*²) of the variation in self-reported aggression (*F*(1,436) = 93.77, *p* < .001). More specifically, negative affectivity (β = −0.11, *p* = .04) was found to be negatively related to aggression, while Disinhibition (β = 0.20, *p* < .001) and antagonism (β = 0.36, *p* < .001) were both positively related to aggression, with antagonism being the strongest predictor. In contrast, detachment (β = 0.08, *p* = .14) and psychoticism (β = 0.04, *p* = .44) did not contribute significantly to the regression model.
Garofalo et al. (2018) [30]	Severity Indices of Personality Problems-Short Form (SIPP-SF) for personality disorder following DSM-5 AMPD	Participants *n* = 376 (all male) consisting of *n* = 138 offenders from the Dutch forensic-psychiatric institution and *n* = 238 control subjects (all male).	138 forensic-psychiatric patients 238 community sample	Violent offenders scored higher on all SIPP-SF domains than the community sample. Correlations of psychopathy and personality functioning impairment between − 0.14 (relational capacities) and − 0.51 (social concordance) for CM, − 0.29 (relation capacities) and − 0.66 (self-control) for violent offenders and − 0.34 (identity integration) and − 0.56 (social concordance) for community sample.
Griffin et al. (2018) [31]	Personality Inventory for DSM-5 (PID-5)	This study involved the participation of 450 (males = 234, females = 216) community-dwelling adults, with an oversampling of individuals who had a history of involvement in the legal system.	450 participants with criminal history	Correlations between PID-5 violent crime and impulsivity (*r* = .13, *p* < .01) and risk taking (*r* = .23, *p* < .001), but not distractibility or irresponsibility. Property delinquency was associated with all PID-5 measures (all r’s > 0.14, all p’s < 0.01),
Hutsebaut et al. (2021) [32]	Semi-structured Interview for DSM-5 Personality Functioning (STiP-5.1), Level of Personality Functioning Brief Form 2.0 (LPFS-BF 2.0)	Participants were recruited by a staff member of the forensic facility. Patients with an intellectual disability or who were in the acute phase of a psychotic disorder were excluded. Non-clinical participants were included in the study (males = 2, females = 16). Participants from the clinical sample were 80 treatment seeking adults (males = 27, females = 53).	98 participants (80 forensic-psychiatric patients). Previously assessed community and clinical samples were used as a reference group.	Independent samples t-tests showed a significant difference on the STiP 5.1 total score between the participants from the community (*M* = 0.56, *SD* = 0.51) and the forensic patients (*M* = 2.60, *SD* = 0.89; *t*(46) = − 8.85, *p* < .001, *d* = 2.70). No significant differences were found between the clinical (*M* = 2.63, *SD* = 0.66) and the forensic sample (*t*(108) = − 0.16, *p* = .874, *d* = 0.04).
Ishfaq & Kamal (2023) [33]	Personality Inventory for DSM-5 Short Form (PID-5 SF) Level of Personality Functioning Brief Form 2.0 (LPFS-BF 2.0)	The sample included 552 convicts incarcerated in all nine Central Jails of Punjab, Pakistan. Convicts are offenders who are sentenced by courts for the crime they have committed. Their major crimes are stealing, robbery, murder, rape, kidnapping, narcotics, fighting and illegal weapon, and white-collar crimes.	552 offenders in prison	Convicts who were sentenced for the offense of drug intake, smuggling, or any other drug-related offense scored high on detachment, disinhibition, Psychoticism as compared to convicts who had committed white collar crimes, murder, or kidnapping (*F*(1,531) = 5.56, *p* < .001, ƞ² = 0.05). Post hoc results showed that convicts imprisoned due to narcotics scored high on Detachment as compared to convicts imprisoned due to offenses like corruption and forgery (mean difference = 0.45, *p* = .012). Convicts imprisoned due to narcotics also scored high on disinhibition as compared to convicts imprisoned due to murder (mean difference = 0.28, *p* = .010) and kidnapping (mean difference = 0.44, *p* = .016). Psychoticism was also reported high among addicts as compared to kidnapers (mean difference = 0.44, *p* = .021). Convicts with previous personal criminal record scored high on detachment (*t* (538) = 2.23, *p* = .026), disinhibition (t (539) = 4.07, *p* < .001), antagonism (*t* (540) = 4.48, *p <* .001), and psychoticism (*t* (541) = 2.47, *p* = .014).
Kuzmickaitė et al. (2019) [34]	The Personality Inventory Short Form (PID-5-SF)	The invitation to participate in the study was extended to all male prisoners sentenced to 3–4 years for minor to moderate crimes where the remainder of their prison sentence was greater than 1 year.	100 offenders in prison	Traits of personality dysfunction were more prevalent in the ADHD prisoners subgroup than in the non-ADHD prisoners group (χ² = 12.617 for negative affectivity, χ² = 10.277 for disinhibition, χ² = 5.876 for antagonism, and χ² = 9.684 for psychoticism, *p* < .05). Multidimensional logistic regression analyses predicted the presence of ADHD symptoms with 88% accuracy (model suitability – determination ratio *R²* = 0.679).
Niemeyer et al. (2022) [35]	The Personality Inventory Forensic Faceted Brief Form (PID-5-FFBF)	Data was collected from *n* = 199 (all male) inmates from a German prison (42.7% convicted for violent offence, 32.7% convicted for sex offence)	199 offenders in prison	Partial correlations of institutional misbehavior with negative affectivity 0.16–0.34, detachment 0.25, antagonism 0.17–0.51, disinhibition 0.14–0.55 and psychoticism 0.25–0.50. Beta coefficients for institutional misbehavior with antagonism 0.16–0.37, disinhibition 0.23–0.37, detachment 0.16 and psychoticism 0.17. Partial correlations of Risk of reoffending with antagonism 0.19–0.24, disinhibition 0.22–0.24 and detachment 0.20. Beta coefficients for Risk of reoffending with antagonism 0.15–0.23, disinhibition 0.30–0.32, and detachment 0.16.
Sellbom et al. (2015) [26]	The Personality Inventory for DSM-5 (PID-5)	The community sample included 140 males and 100 females residing in central Alabama. They were recruited based on advertisements seeking individuals with subclinical psychopathic traits, described as “adventurous, fearless, charming, and carefree people who’ve led exciting lives.” The prison sample consisted of 160 male inmates recruited from a medium-security prison in Kentucky.	240 community sample 160 offenders in prison	Correlations between PID-5-disinhibition and psychopathy (PPI-R) scale for Boldness (*r* = − .21), as well as psychopathy scale for disinhibition (*r* = − .80) for the prison sample, but not the community sample (*R*² = 0.65). Antagonism and detachment were not significantly different between prison and community sample.
Somma et al. (2021) [37]	Personality Inventory for DSM-5 (PID-5)	The sample consisted of 82 adult male inmates serving sentences for sexual offending (75% involving children under 14 years) in Northern, Central, and Southern Italy. Participants were required to be adults (18 years or older), convicted for sexual offenses, with no prior convictions for nonsexual crimes, and without additional nonsexual crime convictions.	82 offenders in prison	Pearson correlations for comparisons of risk assessment (HCR-3) and PID-5 traits were significant for Negative affectivity (Hostility: *r* = .28, *p* < .05, effect size *d* = − 0.30; restricted affectivity: *r* = .34, *p* < .01, effect size *d* = 0.02), detachment (intimacy avoidance: *r* = .34, *p* < .01, effect size *d* = 0.24), antagonism (attention seeking: *r* = .23, *p* < .05, effect size *d* = − 0.19; Callousness: *r* = .27, *p* < .05, effect size *d* = − 0.04), antagonism (manipulativeness: *r* = .22, *p* < .05, effect size *d* = − 0.16), disinhibition (distractibility: *r* = .23, *p* < .05, effect size *d* = − 0.03; impulsivity: *r* = .31, *p* < .01, effect size *d* = − 0.21; risk taking: *r* = .33, *p* < .01, effect size *d* = − 0.18; irresponsibility: *r* = .38, *p* < .001, effect size *d* = 0.19), and psychoticism (eccentricity: *r* = .29, *p* < .01, effect size *d* = − 0.19). These results indicate that these personality traits may play a major role as risk factors for general violence in male adult sexual offenders.
Velotti et al. (2021) [38]	Personality Inventory for DSM-5 (PID-5)	The study included 327 participants divided into two groups. The offender group consisted of 118 Italian men convicted for violent crimes, recruited from prisons in the Latium and Liguria regions. Convenience sampling was used to recruit these participants through educational service operators in the jails. The control group comprised 209 males recruited from the general population, with community participants being recruited by psychology students in their courses.	118 offenders in prison 209 community sample	Antagonism directly and significantly impacts on aggression. Increased disinhibition, as well as increased negative affectivity seem to be linked to lower reflective functioning. The uncertainty about mental states correlated positively with aggression (*r* = .42, *p* < .01) and pathological personality measures (antagonism: *r* = .19, *p* < .05; disinhibition: *r* = .35, *p* < .01; detachment: *r* = .33, *p* < .01; negative affectivity: *r* = .39, *p* < .01; total score: *r* = .42, *p* < .01).
Wygant et al. (2016) [39]	Personality Inventory for DSM-5 (PID-5)	The study included a sample of 200 male inmates who were recruited from Northpoint Training Center, a medium-security prison located in central Kentucky. Inmate participants were recruited either from their dormitories or through the use of recruitment flyers.	200 offenders in prison	Anxiousness and attention seeking contributed incrementally to the prediction of psychopathy domains most strongly linked to boldness (anxiousness *r* = .21, *p* < .01; attention seeking *r* = .22, *p* < .05). Restricted affectivity was consistently correlated with all psychopathy scores (TriPM and PPI-R). However, in the hierarchical regression analyses, they only contributed incrementally to the prediction of fearless dominance.

Note: TriPM: Triarchic Psychopathy Measure [23], PPI-R: Psychopathic Personality Inventory-Revised [24]; HCR-3: The Historical Clinical Risk Management-20, Version 3 [25]; ADHD: Attention Deficit Hyperactivity Disorder

## Overall Descriptives

A total of 610 studies were found with the search string and keywords, whereof a large part had to be excluded based on the exclusion criteria outlined. Here, an inappropriate assessment (i.e., the lack of usage of a dimensional model of personality disorders like DSM-5 AMPD or ICD-11), or the missing examination of a forensic sample, were the most frequent reasons for exclusion (see Fig. 1). Ultimately, 17 studies were selected as eligible.

Most studies were conducted in the United States of America (*k* = 7), followed by the Netherlands (*k* = 3), and Australia, Denmark, Italy (each *k* = 2). Single studies came from Indonesia, Germany, Lithuania, and Pakistan. Sample sizes ranged from 82 to 600 participants (*M* = 255.2, *SD* = 148.5). Most studies (90%) were focused on a description of *Criterion B* of the dimensional conception of personality disorder (versions of the PID-5). Only four of the selected papers involved (additional) consideration of *Criterion A* [26–29]. Eight studies (47%) considered female samples in addition to male samples.

The majority of the included studies report results from offenders in prison (*k* = 13), some of which compared these to a community sample (*k* = 5). Five studies included forensic patient samples as a control group (*k* = 3) or as a single group (*k* = 2).

Seven studies report on group comparisons (offenders in prison versus community and/or forensic-patient sample), three studies investigated associations between PID-5 and measures of psychopathy, three studies examined associations between PID-5 and intramural aggression, five studies focused on criminality or risk of recommitting violent crimes, and three studies investigated other measures of interest with regard to their association with PID-5 domains (e.g., mentalizing, substance abuse, identity dysfunction, emotion regulation, and childhood trauma).

Lower Levels of Personality Functioning of *Criterion A* domains were predominantly higher in offenders in prison, as compared to community or forensic patient samples (*k* = 2 each). Even though the number of studies is small for this facet, the evidence is strong, as one study [29] used a multi-method approach, as compared to mere self-report and found a large mean difference in LPFS levels between community and forensic samples. Associations between maladaptive personality traits (PID-5 domains) of *Criterion B* and measures of interest were found to be higher in prison samples, predominantly for antagonism (*k* = 15) and disinhibition (*k* = 14), but less for negative affectivity or detachment (*k* = 10 respectively), and psychoticism (*k* = 8).

### Group Comparisons

In total, confirmatory of higher personality impairment measures for incarcerated populations are reported by 80% of studies. All studies engaging in group comparisons between an imprisoned and a community sample report higher personality impairments in the prison sample [27–32]. However, two studies only investigated associations to PID-5 characteristics indirectly through intercorrelations with traits of psychopathy [31], or aggression [32].

Results of Adhiatma and Halim [30] revealed significant differences among groups of violent offenders, non-violent offenders, drug offenders, and non-prisoners in several domains, including negative affectivity, antagonism, detachment, disinhibition, and psychoticism. Post hoc analyses further highlighted specific areas of divergence, with violent and drug offenders consistently showing higher average scores compared to non-violent offenders and non-prisoners. These findings underscore the importance of considering personality functioning in the context of different offender groups and highlight potential areas of focus for interventions and treatment.

Bach and Hutsebaut [27] examined the differences in self-functioning and interpersonal functioning between outpatients and incarcerated addicts using the LPFS-BF 2.0 measure. Outpatients demonstrated higher scores on self-functioning, while offenders in prison had slightly higher scores on interpersonal functioning. LPFS-BF 2.0 significantly predicted external correlates, and in some cases, PID-5 scores added to the predictive utility.

The study of Hutsebaut and colleagues [29] found that a forensic sample demonstrated significantly higher personality dysfunctioning (STiP 5.1 total scores) compared to a community sample. However, no significant differences were found between forensic and clinical samples. This study highlights the importance of considering the mode of assessment, as it confirms the discrepancies between self-report and expert-ratings, which may be especially prominent in forensic settings (see limitations).

Garofalo [28] investigated group differences between violent offenders in a sample of forensic-psychiatric patients and compared them to a community sample on levels of self-reported personality functioning and psychopathic traits. Bivariate associations revealed that violent offenders showed higher levels of personality dysfunctions and psychopathy, compared to community participants.

The study of Selbom and colleagues [31] examined the intercorrelations between psychopathy (PPI-Triarchic scales) and their associations with external criteria in community and prison samples. PPI-Boldness was moderately associated with low negative affectivity and detachment, as well as high antagonism and disinhibition. PPI-meanness showed the strongest association with PID-5 antagonism and contributed uniquely to the prediction of narcissistic personality disorder (NPD) and antisocial personality disorder (ASPD). PPI-disinhibition demonstrated large associations with PID-5 disinhibition and uniquely predicted all PID-5 domains. It was also positively correlated with negative affectivity, psychoticism, and detachment. PPI-disinhibition significantly and uniquely predicted antisocial behavior and substance use.

Velotti and colleagues [32] tested the mediating role of mentalizing in the relationship between the three pathological personality domains and aggressiveness in a sample consisting of offenders and non-offenders. Results suggest that antagonism had a direct and significant impact on aggression, while negative affectivity and disinhibition had direct negative effects on reflective functioning.

## Offenders in Forensic-Psychiatric Care or Prison

Concerning studies which focused only on forensic samples [26, 33–41], results are heterogenous, addressing a multitude of psychological constructs, such as maladaptive traits [26], regulative strategies [33], forensically relevant outcomes (e.g., number of arrests [34], aggression/violence [35, 36, 39, 40], or psychopathy [41]. The results of two studies [37, 38] are uninterpretable due to very specific group comparisons that are not informative for the current review (differences in type of crime and Attention Deficit Hyperactivity Disorder (ADHD) vs. non-ADHD offenders in prison).

Even though the study of Bach and Anderson [26] recruited two different samples consisting of prisoners and psychiatric outpatients, the two subsamples were combined in order to ensure sample heterogeneity and circumventing range restrictions. The study aimed to examine the associations between personality impairment measures (LPFS-BF, SASPD) and various external correlates. However, the SASPD should be interpreted with caution, as its convergent and discriminant validity internal consistency is limited and the internal consistency merely acceptable in the clinical and nonclinical samples, while data on forensic samples is lacking. Maladaptive schemas correlated with levels of personality functioning on all scores. Their findings highlight the differential associations and predictive abilities of LPFS-BF and SASPD with external correlates, emphasizing the importance of considering both measures in personality impairment research.

Billen and colleagues [33] examined the longitudinal and cross-sectional relationships between various psychological constructs, including emotion regulation, behavioral regulation, cognitive regulation, childhood trauma, identity dysfunction, antagonism, detachment, negative affectivity, and psychoticism. Longitudinal analyses revealed no substantial change in emotion regulation, or cognitive regulation over time for forensic-psychiatric patients. Cross-sectional analyses revealed that emotion regulation was significantly associated with all outcome variables, behavioral regulation was associated with most outcome variables, and cognitive regulation was associated with antagonism and negative affectivity.

The study of Boland and colleagues [34] examined the associations between childhood maltreatment, maladaptive personality traits, and criminal behavior using a dimensional trait model of personality psychopathology. Childhood maltreatment was positively associated with maladaptive personality domains (especially negative affectivity). The number of adult arrests was significantly associated with personality psychopathology variables.

Results of the studies by Dunne [35, 36] showed that the PID-5-BF positively correlated with self-reported aggression in male imprisoned offenders. Multiple regression analyses revealed that the total score and domain scores predicted variations in aggression, with antagonism being the strongest predictor.

The study of Niemeyer [39] used an adaptation of the PID-5 to assesses maladaptive personal traits in forensic settings (PID-5 Faceted Brief Form; PID-5-FBF) with an exploratory factor analyses suggesting a four-factor solution comprising antagonism, detachment, disinhibited aggression, and insecurity. Antagonism and disinhibited aggression were associated with higher levels of institutional misbehavior and risk for reoffending. Regression analyses including all four domains of the PID-5-FFBF as predictors further supported the relevance of disinhibited aggression in predicting institutional misbehavior and recidivism.

Ishfaq and Kamal [37] examined different types of crime and their relation to the PID-5 in a prison sample. Convicts who were sentenced for the offense of drug-related offenses scored high on detachment, disinhibition, psychoticism as compared to convicts who committed other types of crimes.

Kuzmickaitė and colleagues [38] aimed to assess the prevalence of ADHD symptoms among offenders and examine their association with demographic factors, psychiatric disorders, personality dysfunction, and offense-related variables. Negative affect, antagonism, disinhibition, and psychoticism were more prevalent in the ADHD subgroup.

The study of Somma and colleagues [40] aimed to associate PID-5 trait scale scores with risk assessments commonly used in forensic settings (HCR-20) in a sample of male sexual offenders. Correlations were significant for all five domains of the PID-5, indicating that these personality traits may play a major role as risk factors for general violence in male adult sexual offenders.

The study of Wygant and colleagues [41] compared the associations between the DSM-5 Section III (PID-5) and Section II (SCID-II ASPD) with psychopathy. Psychopathy was positively associated with attention seeking and negatively associated with withdrawal. Additional traits such as grandiosity, restricted affectivity, and submissiveness contributed incrementally to the prediction of specific psychopathy domain scores. Identity, self-direction, empathy, and intimacy correlated moderately to highly with psychopathy scores.

## Discussion

This systematic review analyzes the current empirical literature on the dimensional approaches of the DSM-5 AMPD and ICD-11 to assess levels of personality functioning and personality disorder severity in criminal offenders. Due to the challenges diagnostics and treatments it seems important to focus on precise treatment indications and prognosis. Therefore, the question which added value dimensional approaches such as the ones implemented in DSM-5 AMPD or in ICD-11 bring to forensic settings is of high relevance.

Our systematic search revealed a decent number of studies investigating DSM-5AMPD *Criterion B* in predominantly male forensic inmates mostly using a version of the PID-5. The most consistent result of these studies were high levels in facets of the trait domains antagonism [26, 27, 30–40], disinhibition [26, 27, 30, 35–40, 42], and negative affectivity [30, 34, 37–41] to be elevated in offenders. The currently limited number of studies which additionally assessed *Criterion A*, most often with the LPFS, found impairments on subscores [26], total scores [28, 29], or lower scores on interpersonal functioning [27] in offenders than community samples with large differences between forensic and community samples (two LPFS severity levels). Three studies reported additional effects of aggression in offenders with regards to the expression of *Criterion B* [32, 35, 36], that is, the PID-5 total score and domain scores, especially antagonism, were positively correlated with self-reported aggression.

Since the ICD-11 is still new and instruments have only recently been developed with translations or psychometric criteria being less available than for those assessing DSM-5 AMPD, it seems plausible that the current review finds more studies on the latter. Even though data from clinical samples suggest that especially self-functioning may represent an important predictor for treatment response, more (longitudinal) research on *Criterion A* (together with *Criterion B*) in forensic samples is urgently needed [43, 44]. With regard to *Criterion A*, available literature in forensic samples is still limited and does not allow for extensive interpretation, although impairments in the domains of identity (self-functioning) and empathy (interpersonal functioning) have been suggested [45] with the majority of results from interviews pointing towards two LPFS severity levels difference between forensic and community samples.

Based on the presented number of studies, findings on *Criterion B* might be regarded as more robust despite considerable heterogeneity in study designs, populations, measures, and individual results. The most consistent finding across studies was higher scores of antagonism and disinhibition in forensic samples than previously measured in community samples. In fact, the empirical relations between personality traits and antisocial behavior have shown that traits related to antagonism and disinhibition are implicated in a variety of antisocial behaviors [46, 47]. Longitudinal evidence also suggests that antagonistic traits predict antisocial behavior both concurrently and later in life [48, 49]. These studies provide evidence for the presence and relevance of antagonism and disinhibition in offenders and suggest that these traits may be associated with aggressive behavior and other psychopathy-related criteria [47]. Results were less consistent with regard to negative affectivity. Clinical studies found high levels of facets of negative affectivity in individuals with Borderline Personality Disorder [50], but not with Antisocial Personality Disorder [51], while facets of antagonism and disinhibition were reported in both disorders in clinical samples and offenders [41, 45].

Associations between levels of personality functioning and psychopathy was also investigated by some of the current studies [28, 31, 41], challenging the classical psychopathy construct by Hare in designating psychopaths as a discrete subgroup of individuals [52]. The most commonly utilized and validated procedure to evaluate psychopathy remains to be the revised Psychopathy Checklist (PCL-R; [53]) for forensic psychological and psychiatric risk assessments, although there have been criticisms [54] and its dimensionality has been a topic of debate among researchers [55]. Even though the PCL-R consists of several factors that capture different aspects of psychopathy, including interpersonal, affective, lifestyle, and antisocial aspects, it has been suggested that a consideration of independent components of psychopathy along a continuous scale could improve assessments and treatments targeting psychopathy [56].

Even in light of the introductory remarks about the concerns of forensic psychiatrists in applying dimensional assessments for personality disorders, there are several advantages that should be considered. *Criterion A* reflects the dimensional nature of personality differences and makes normative ideas about undisturbed personality explicit, while still allowing for an assessment of severity and hybrid use of previous treatment manuals. *Criterion B* has a good empirical foundation, thereby establishing a connection to basic research, and thoroughly covers the spectrum of possible personality problems. It further describes problems on different levels of abstraction and allows for creating an individual personality profile [57, 58]. Although the heterogeneity of the selected studies renders it difficult to draw clear conclusions, findings suggest that these dimensional approaches allow for a precise description of predominant trait patterns across categorical boundaries. A direct comparison between categorical and dimensional models is needed to assess the added predictive value of these models for forensic populations. The implementation of targeted interventions based on trait profiles in offenders might be an important step in future to improve tailored treatment and prognoses.

### Limitations and Final Remarks

Besides the still limited number of studies (particularly for ICD-11) and their heterogeneity in quality and quantity, our own selection process might have been prone to bias due to its specific focus and the application of rather strict in- and exclusion criteria. Furthermore, not all studies report group comparisons with a healthy control population, but rather refer to previously assessed healthy samples. This further limits interpretability and comparability of the selected studies.

Another noteworthy limitation of this review is the fact that most studies rely on self-report. The concern about the validity of self-report measures has been addressed previously and may be especially relevant in forensic samples. The multi-methodological approach of the study by Hutsebaut and colleagues [29] substantiates this problem, as the authors find a clear association between a clinical patient’s self-assessment of his or her personality features and the expert-based assessment, yet large discrepancies in the forensic sample between self-report and expert ratings. The study thereby identifies the need to design and test assessment instruments within this context instead of generalizing findings obtained in regular mental health care samples.

Taken these caveats in account, the current review suggests that an additional dimensional conceptualization of personality functioning provides grounds for improvements concerning the assessment within a forensic-psychiatric context, as it enables individualization, therefore allowing for a more precise treatment. However, treatment planning is only a secondary step in forensic assessments, as the primary focus rather lies in the establishment of *severe mental abnormality* as entry requirement to decide on the placement of an offender in countries like Germany [59]. Given the decrease of the diagnostic threshold through dimensional assessments, even more attention must be paid to the distinction between clinical diagnosis and legal diagnosis [59]. The idea of more precision with more complex assessment through dimensionality seems promising, yet not empirically investigated and potentially an important next step for future research.

A more general limitation to using dimensional assessments to forensic samples is the fact that some items in the PID-5, for example, cannot apply to or be answered freely by incarcerated individuals (e.g., “I often forget to pay my bills”). Some items may differ with regard to the situation of the questioned individual (e.g., the response to “run away” entails different meaning for imprisoned offenders, or the willingness to make friends or see family may differ in an intra- as compared to an extramural context). For these reasons, the work of Niemeyer and colleagues [39] in developing a modified version of the questionnaire, applicable to forensic populations, and with an advised reassessment policy after two years, is highly valuable and necessary.

Even though an effort was made to differentiate between offenders in prison and those in psychiatric care, we acknowledge the fact that the term “forensic setting” has not differentiated respective groups of offenders adequately in previous research, which may also influence our reviewed results. Therefore, it is of note that prevalence and distribution of offenders may differ according to the respective setting. More distinct differentiations should be considered in future research [60].

## Conclusion

In conclusion, the discussed dimensional models have several advantages for the assessment and especially the treatment of personality disorders. The reviewed studies suggest that these advantages convey to forensic settings, yet the practical relevance especially in comparison to traditional categorical models and the triadic model as foundation for prognosis remains unanswered. It is therefore important to consider the clinical, as well as the forensic and legal utility of dimensional assessments in forensic settings. With regards to its clinical utility, the DSM-5 AMPD [61, 62], as well as the ICD-11 [63] demonstrated its ability to conceptualize patients with individually distinct personality functioning and severity in a meaningful and comprehensible way that would be helpful to both therapist and patient. Concerning its forensic utility, there is evidence to suggest that they may be useful in predicting patient outcomes and capturing certain aspects of personality pathology, as well as improving individual and sustainable diagnoses to help tackle specific issues of treating perpetrators with specific personality profiles. The findings of this systematic review provide initial evidence for the validity and utility of DSM-5 AMPD and ICD-11 in forensic settings, which nevertheless need to be confirmed by future research into multiple directions to broaden the view on this topic.

## Data Availability

No datasets were generated or analysed during the current study.
